# Sleep patterns and sleep disorders in pregnant women with preeclampsia: a systematic review and meta-analysis

**DOI:** 10.61622/rbgo/2026rbgo54

**Published:** 2026-08-20

**Authors:** Rebeca Lundii Oliveira Pereira, Naila Giovana Ferraz de Oliveira, Celia Regina Santos Cavalcanti, Luisa Six Maeda, Nelson Sass

**Affiliations:** 1 Universidade Federal de São Paulo Escola Paulista de Medicina São Paulo SP Brazil Escola Paulista de Medicina, Universidade Federal de São Paulo, São Paulo, SP, Brazil.

**Keywords:** Preeclampsia, Pregnancy women, Sleep apnea syndrome, Sleep disorders, Sleep wake disorders, Pregnancy outcome

## Abstract

**Objective:**

Preeclampsia (PE) is a hypertensive disorder of pregnancy with diverse and poorly understood risk factors. Hormonal changes during this period affect sleep, raising the question of whether sleep disturbances may be related to PE. This review aimed to investigate the prevalence of sleep disturbances among pregnant women diagnosed with PE.

**Methods:**

We included observational studies and clinical trials assessing sleep by any method. Exclusion criteria were studies on PE without sleep assessment, systematic reviews, editorials, animal studies, and unavailable full texts. Studies were retrieved from Virtual Health Library, MEDLINE and Embase using the descriptors "preeclampsia" and "sleep" with additional manual searches. The last search was conducted on December 10, 2024. Screening and data extraction were performed independently by two reviewers. Risk of bias was assessed using the Joanna Briggs Institute checklist. Random-effects models were used to synthesize prevalence as proportions with 95% confidence intervals (CIs).

**Results:**

Twenty-one studies were included from 2,075 initially identified. The pooled prevalence of sleep disturbances in PE was 46% (95% CI [0,19 - 0,76]), p=0,823. Stratified analysis identified two categories: qualitative disturbances, with prevalence of 50% (95% CI [0.31–0.70]); and sleep-disordered breathing with prevalence of 43% (95% CI [0.10–0.83]).

**Discussion:**

This meta-analysis found an estimated 46% prevalence of sleep disturbances in preeclampsia; however, the evidence is of very low quality (GRADE), due to extreme heterogeneity and uncontrolled risk of bias.

**PROSPERO:** CRD42024557766

## Introduction

Preeclampsia is one of the leading causes of maternal and perinatal morbidity and mortality worldwide, accounting for approximately 76,000 maternal deaths and 500,000 fetal deaths annually, particularly in low- and middle-income countries.^(1)^ It is a pregnancy-specific hypertensive syndrome that affects between 2% and 8% of pregnant women globally and is characterized by the onset of arterial hypertension and signs of target-organ damage, such as renal or hepatic dysfunction, after the 20th gestational week.^(2)^ In Brazil, this condition represents the main cause of maternal mortality, with more than 300 deaths per year.^(3)^ Despite advances in prenatal care, early identification and management of preeclampsia remain challenging, especially in regions with limited access to specialized health services.^(4)^

Multiple factors have been implicated in the pathophysiology of preeclampsia, including immunological alterations, endothelial dysfunction, and genetic and environmental influences, as well as preexisting conditions such as obesity and cardiovascular disease.^(5)^ However, the exact mechanism that triggers the disorder is still not fully understood. The complexity of the syndrome and the variability among cases suggest that multiple elements contribute to its development and severity.^(6)^

Studies indicate that pregnancy itself is associated with significant changes in sleep patterns, with a high prevalence of disorders such as insomnia, obstructive sleep apnea, and sleep fragmentation.^(7)^ Poor sleep quality during pregnancy has been associated with adverse obstetric outcomes, including preterm birth, gestational diabetes, and hypertension.^(8)^ Evidence in the literature supports this association, demonstrating that the use of nasal CPAP in pregnant women with hypertension and chronic snoring can improve blood pressure control and perinatal outcomes.^(9)^

Furthermore, sleep disorders are associated with an increased risk of preeclampsia. Multiple high-quality studies and meta-analyses demonstrate that sleep disorders—including sleep-disordered breathing (SDB), obstructive sleep apnea (OSA), insomnia, poor sleep quality, and abnormal sleep duration—are independent risk factors for the development of preeclampsia during pregnancy.^(10-14)^

The Society for Maternal-Fetal Medicine specifically recognizes sleep-disordered breathing as a validated risk factor for preeclampsia, with adjusted odds ratios close to 2 for SDB in early and mid-pregnancy.^(13)^ Meta-analyses show that sleep disorders may increase the likelihood of preeclampsia by nearly sevenfold, with a clear dose–response relationship between the frequency and severity of sleep-related symptoms.^(10-11)^ Both short sleep duration (<7 hours) and long sleep duration (>9 to 10 hours), as well as reduced sleep efficiency and habitual snoring, are associated with higher blood pressure and increased risk of preeclampsia.^(14-16)^

Sleep disorders may contribute to preeclampsia through increased sympathetic activation, impaired vascular function (including increased arterial stiffness) and adverse metabolic effects, with body mass index acting as a partial mediator.^(17-19)^ Disordered breathing during sleep, in particular, is associated with increased arterial stiffness and greater risk when accompanied by hypersomnolence.^(17-18)^

Although evidence indicates that sleep disorders increase the risk of preeclampsia, important gaps remain in the literature. Most studies evaluate sleep as a predictor of disease development, but few investigate the prevalence of sleep disorders among women already diagnosed with preeclampsia. Moreover, existing studies use heterogeneous assessment methods. This lack of systematic synthesis hampers understanding of the true burden of these disorders in this population and limits the development of targeted screening and management strategies.

Therefore, the aim of this study was to investigate the prevalence of sleep disorders among pregnant women diagnosed with preeclampsia, including subjective sleep quality and respiratory disturbances, in order to better elucidate the relationship between sleep quality and the development or worsening of preeclampsia. Understanding the prevalence of these disorders may further guide clinical practice and inform future research on maternal health.

## Methods

The study protocol is available in the PROSPERO registry (International Prospective Register of Systematic Reviews) under the registration number CRD42024557766. This review was conducted in accordance with the Cochrane Handbook for Systematic Reviews of Interventions^(20)^ and reported following the Preferred Reporting Items for Systematic Reviews and Meta-Analyses (PRISMA 2020) guidelines.^(21)^

The research question was structured using the PICO framework as follows: Population (P): pregnant women with a confirmed diagnosis of preeclampsia (based on ACOG or ISSHP criteria); Exposure/Intervention (I): presence or assessment of sleep disorders (qualitative disturbances such as insomnia and excessive daytime sleepiness; respiratory disturbances such as obstructive sleep apnea and snoring); Comparator (C): not applicable, as prevalence was assessed; Outcomes (O): Primary – prevalence of sleep disorders in women with preeclampsia; Secondary – specific types of disorders and degrees of severity. Study design (S): observational studies (cross-sectional, case-control, and cohort studies) and clinical trials, with no restrictions regarding date, language, or publication status.

Articles were retrieved from major databases, including the Latin American and Caribbean Health Sciences Literature (LILACS, via the Virtual Health Library), Medical Literature Analysis and Retrieval System Online (MEDLINE, via PubMed), and Embase (via Elsevier), as well as through manual screening of the reference lists of selected studies.

### Search strategy

The search strategy was developed using Medical Subject Headings (MeSH) and their related entry terms. The search strategy combined controlled vocabulary (Medical Subject Headings – MeSH and Emtree terms) and free-text terms related to "preeclampsia" and "sleep," using Boolean operators (AND, OR). The full search strategies for each database, including all search terms, controlled vocabulary, and combinations, are provided in the Supplementary Material (Table 1S). The last search was conducted on December 10, 2024.

### Eligibility criteria

Studies were included if they: (a) investigated the association or prevalence of sleep disorders in pregnant women diagnosed with preeclampsia (≥ 18 years); (b) used an observational design or clinical trial; and (c) assessed sleep using any method (validated questionnaire, polysomnography, or actigraphy). Exclusion criteria comprised: (a) studies on preeclampsia without any assessment of sleep; (b) systematic reviews, editorials, commentaries, animal studies, and studies for which full-text access was unavailable.

### Study selection

Study selection was conducted using the Rayyan platform. All reviewers independently screened titles and abstracts and assessed full texts based on the eligibility criteria. Duplicates were removed prior to screening. Discrepancies were resolved through discussion and consensus. Data extraction was performed independently and included year of publication, country, study design, sample size, mean age, method of sleep assessment, and prevalence of sleep disorders.

### Statistical analysis

Prevalence estimates were extracted as proportions and pooled using a random-effects model (DerSimonian–Laird), weighted by inverse variance. A random-effects model was pre-specified due to the expected methodological and clinical heterogeneity. Proportions were transformed into log event rates before conducting the aggregate meta-analysis. Effect sizes were reported with 95% confidence intervals and two-tailed p-values (α = 0.05). Statistical heterogeneity was assessed using the I^2^ statistic and Cochran's Q test. Values of I^2^ > 50% were interpreted as indicative of substantial heterogeneity. All quantitative analyses, including heterogeneity metrics and figure generation, were performed using ProMeta 3 software.

### Bias assessment

Risk of bias was independently assessed by two reviewers using the Joanna Briggs Institute (JBI) Critical Appraisal Checklist. Any disagreements regarding the evaluation criteria were reviewed jointly until consensus was reached. Publication bias was assessed using: (1) visual inspection of the funnel plot, (2) Egger's test, (3) the trim-and-fill method to estimate missing studies, and (4) Begg and Mazumdar's test.

The findings of each included study were summarized narratively in a characteristics table, including population, outcomes, and main results, enabling exploration of clinical heterogeneity prior to quantitative synthesis.

## Results

A total of 2,075 potentially eligible articles were identified: 2,031 from databases (MEDLINE, n = 1.804; Embase, n =225; LILACS, n = 2) and 44 via citation searching. Off these, 143 met criteria for full-text assessment. Ultimately, 21 studies were included in the systematic review, 20 of which were included in the meta-analysis (Figure 1).

**Figure 1:**
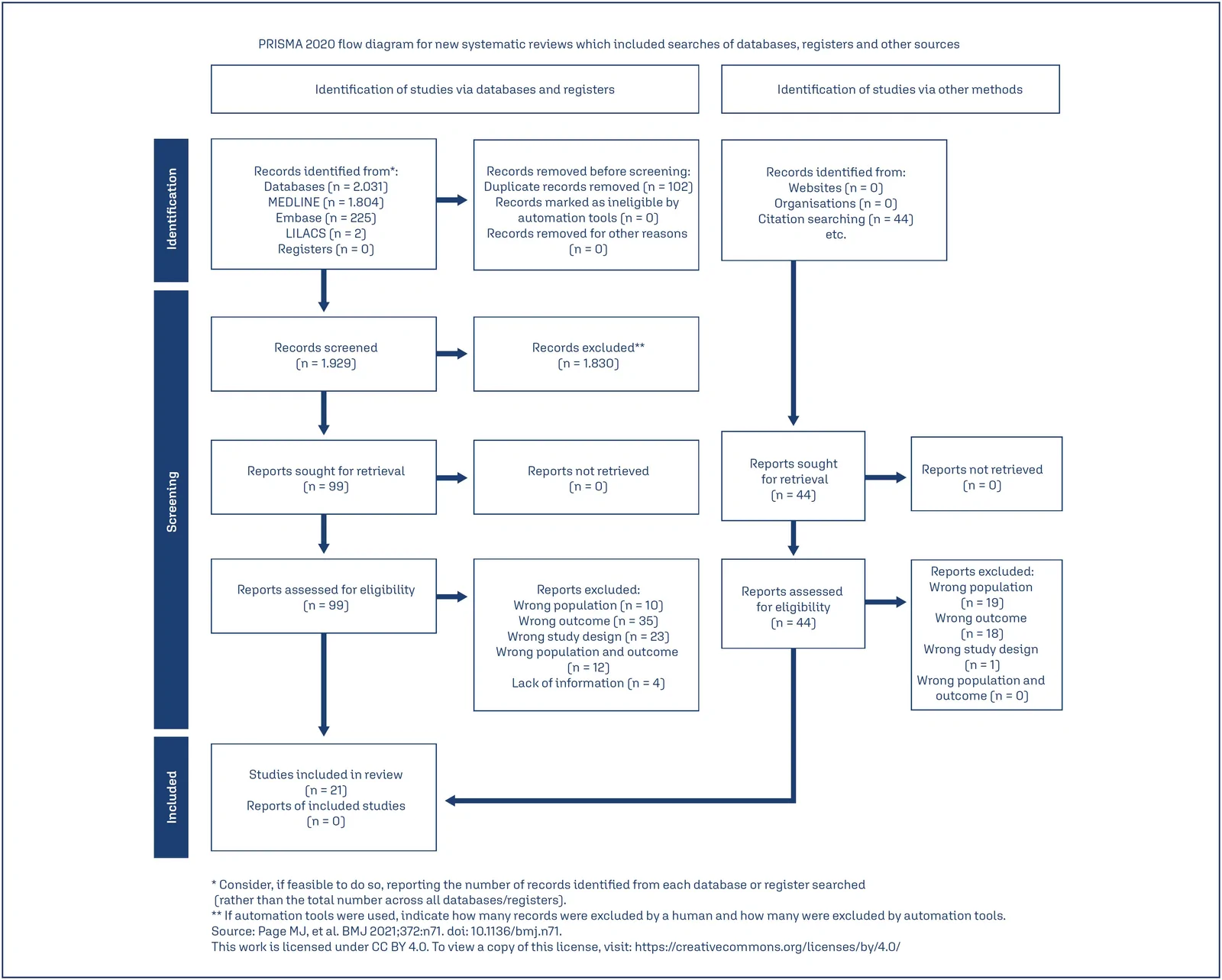
PRISMA 2020 flowchart: study selection process for systematic review on sleep disturbances and preeclampsia

Twenty-one articles investigating the prevalence of sleep-related disturbances in women with preeclampsia were included, encompassing both subjective sleep quality and the presence of respiratory sleep disorders. Most studies assessed sleep quality and respiratory disturbances simultaneously. Therefore, the analysis was organized into two distinct categories: (1) sleep quality, including insomnia and subjective sleep perception; and (2) sleep-disordered breathing, such as habitual snoring, obstructive sleep apnea (OSA), airflow limitation, and reduced REM sleep. Accordingly, two separate meta-analytic plots were generated to represent these domains individually. The characteristics of the 21 included studies are summarized in Table 1, including study design, sample size, population characteristics, sleep assessment methods, and reported prevalence estimates.

**Table 1:** Characteristics of 21 included studies investigating the association between preeclampsia and sleep disturbances in pregnant women

	Country	Study design	n	Age	Outcome category	Assesed characteristic	Methods	Prevalence of the assed characteristic
Ursavas A., et al.^(22)^, 2008	Turkey	Case-control	30	27.3 ± 6.5	Sleep-disordered breathing	Habitual snoring (>3 times per week)	Self-developed questionnaire	6/30 (Habitual snoring)
Izci B., et al.^(23^ 0, 2005	United Kingdom	Case-control	82	30 ± 6	Sleep-disordered breathing Subjective sleep quality	Habitual snoring (>3 times per week) and sleepiness (ESS >10)	Self-developed questionnaire and Epworth Sleepiness Scale (ESS)	48/82 (Habitual Snoring); 12/82 (Excessive daytime sleepiness)
Malhamé I., et al.^(24)^, 2022	EUA	Retrospective cohort	71.159	31 ± 6	Sleep-disordered breathing	Obstructive sleep apnea	Hospital records (2010 to 2014)	270/71.159 (Obstructive sleep apnea)
Wilson DL., et al.^(25)^, 2020	Australia	Case-control	17	31.0 (30.0, 34.0)	Sleep-disordered breathing	Obstructive sleep apnea	Polysomnography	7/17 (Obstructive sleep apnea)
Savka R., et al.^(26)^, 2023	Ukraine	Case-control	50	29.1 ± 3.4	Subjective sleep quality	Sleep disorders (insomnia, awakenings, morning fatigue)	Self-developed questionnaire	44/50 (sleep disorders)
Zhu J. et al.^(15)^, 2024	China	Prospective cohort	97	25-35	Sleep-disordered breathing Subjective sleep quality	Habitual snoring (>1 time per week) and sleep quality (PSQI >5)	Self-reported snoring and Pittsburgh Sleep Quality Index	9/97 (Habitual snoring); 30/97 (Poor sleep quality)
Ugur MG., et al.^(27)^, 2012	Turkey	Cross-sectional	37	28.1 ± 6.2	Sleep-disordered breathing	Snoring	Berlin Questionnaire	19/37 (Snoring)
Khazaie H., et al.^(28)^, 2013	Iran	Cross-sectional	102	29.3 ± 6.9	Sleep-disordered breathing Subjective sleep quality	Insomnia, snoring, apnea, sleep fragmentation, and daytime sleepiness	Global sleep assessment questionnaire (GSAQ)	27/102 (Insomnia); 22/102 (Snoring);
Izci B., et al.^(29)^, 2003	United Kingdom	Cross-sectional	37	29 ± 5	Sleep-disordered breathing	Snoring (>1 time per week)	Self-developed questionnaire	28/37 (Snoring)
Yin TT., et al.^(30)^, 2008	United Kingdom	Cross-sectional	33	29 ± 6.6	Sleep-disordered breathing Subjective sleep quality	Habitual snoring, and daytime sleepiness (ESS >10)	Epworth Sleepiness Scale (ESS) and oxygen monitoring	4/33 (Habitual snoring); 5/33 (Daytime sleepiness)
Georgiou N., et al.^(31)^, 2019	Greece	Retrospective cohort	22	28.27 ± 8.78	Subjective sleep quality	insomnia (AIS>6)	Athens Insomnia Scale (AIS)	8/22 (Insomnia)
Kordi M., et al.^(32)^, 2015	Iran	Case-control	151	29,5 ± 4,1	Subjective sleep quality	Sleep quality (PSQI >5)	Pittsburgh Sleep Quality Index (PSQI)	138/151 (Poor sleep quality)
Ekholm EM., et al.^(33)^, 1992	Finland	Comparative cross-sectional	9	20-34	Subjective sleep quality	Sleep quality and nocturnal motor activity	Self-developed questionnaire	3/9 (Poor sleep quality);
Blyton DM., et al.^(34)^, 2013	Australia	Case-control	20	33.6 ± 6.6	Sleep-disordered breathing Subjective sleep quality	Sleep quality and sleep-disordered breathing (AHI>10 and airflow limitation)	Polysomnography	9/20 (Poor sleep quality); 13/20 (Sleep disordered breathing)
Takmaz T., et al.^(35)^, 2020	Turkey	Prospective cohort	104	25-36	Subjective sleep quality	Sleep quality (PSQI >5)	The Pittsburg sleep quality questionnaire	91/104 (Poor sleep quality)
Facco FL., et al.^(36)^, 2013	EUA	Case-control	20	19 - 33	Sleep-disordered breathing	Sleep-disordered breathing (AHI>10) and sleep quality	Watch-PAT100 and author-developed question about non-restorative sleep	3/20 (Sleep disordered breathing); 16/20 (Non-restorative sleep)
Keshavarzi F., et al.^(37)^, 2018	Iran	Case-control	40	30,8 ± 6,5 anos	Sleep-disordered breathing	Obstructive Sleep Apnea (mild, moderate, and severe AHI)	SOMNOwatch plus Respiratory Screener	39/40 (Obstrtuctive sleep apnea)
Yinon D., et al.^(38)^, 2006	Israel	Case-control	17	30±7	Sleep-disordered breathing	Sleep-disordered breathing	Watch-PAT100	15/17 (Sleep-disordered breathing)
Connolly G., et al.^(39)^, 2001	Irlanda	Case-control	15	28.0±2.3	Sleep-disordered breathing	Inspiratory flow limitation	Polysomnography	-
Bachour A., et al.^(40)^, 2008	Finland	Comparative cross-sectional	15	33.7 ± 6.3	Sleep-disordered breathing Subjective sleep quality	Increased snoring frequency, habitual snoring (>2 times per week), and sleep quality	Self-developed questionnaire	10/15 (Increased Snoring Frequency); 6/15 (Habitual snoring); 4/15 (Poor sleep quality)
Blyton DM., et al. ^(41)^, 2004	Australia	Clinical trial	24	33 ± 6	Sleep-disordered breathing	Upper airway flow limitation	Polysomnography	24/24 (Upper airway flow limitation)

### Study characteristics

Ten studies employed a case–control design, six were cross-sectional, two were retrospective cohorts, two were prospective cohorts, and one was a clinical trial. The studies were published between 1992 and 2023 and conducted across several countries, including the United Kingdom, Turkey, Iran, China, the United States, Australia, Finland, Greece, Israel, Ukraine, and Ireland. Sample sizes varied widely, ranging from small cohorts (n = 9) to large-scale population studies (n > 70,000). There was marked heterogeneity regarding the sleep disturbances assessed and the methods used. Most studies investigated habitual snoring, sleep–respiratory symptoms, or poor sleep quality through self-reported questionnaires (Berlin, PSQI, ESS, AIS, or author-developed instruments). A smaller number used objective assessments, including polysomnography, portable cardiorespiratory devices (Watch-PAT100, SOMNOwatch), or oxygen and airflow monitoring. Reported prevalence estimates varied considerably across studies, reflecting differences in diagnostic criteria and measurement instruments. Studies utilizing instrumental assessments tended to identify higher proportions of respiratory sleep disturbances, whereas questionnaire-based studies more frequently reported symptoms such as insomnia, sleepiness, and poor sleep quality. The study by Connolly G. (2001) was included only in the qualitative synthesis, as it did not provide prevalence data compatible with inclusion in the meta-analyses. The pooled analysis estimated an overall prevalence of 0.46 (95% CI [0.19–0.76]) (Figure 2). The confidence interval was wide and consistent with p-value of 0.823 in the two-tailed test of the pooled estimate, which reflects substantial uncertainty. Heterogeneity across studies was extremely high (I^2^ = 99.27%), indicating considerable variability in effect sizes (range: 0.00–0.97).

**Figure 2:**
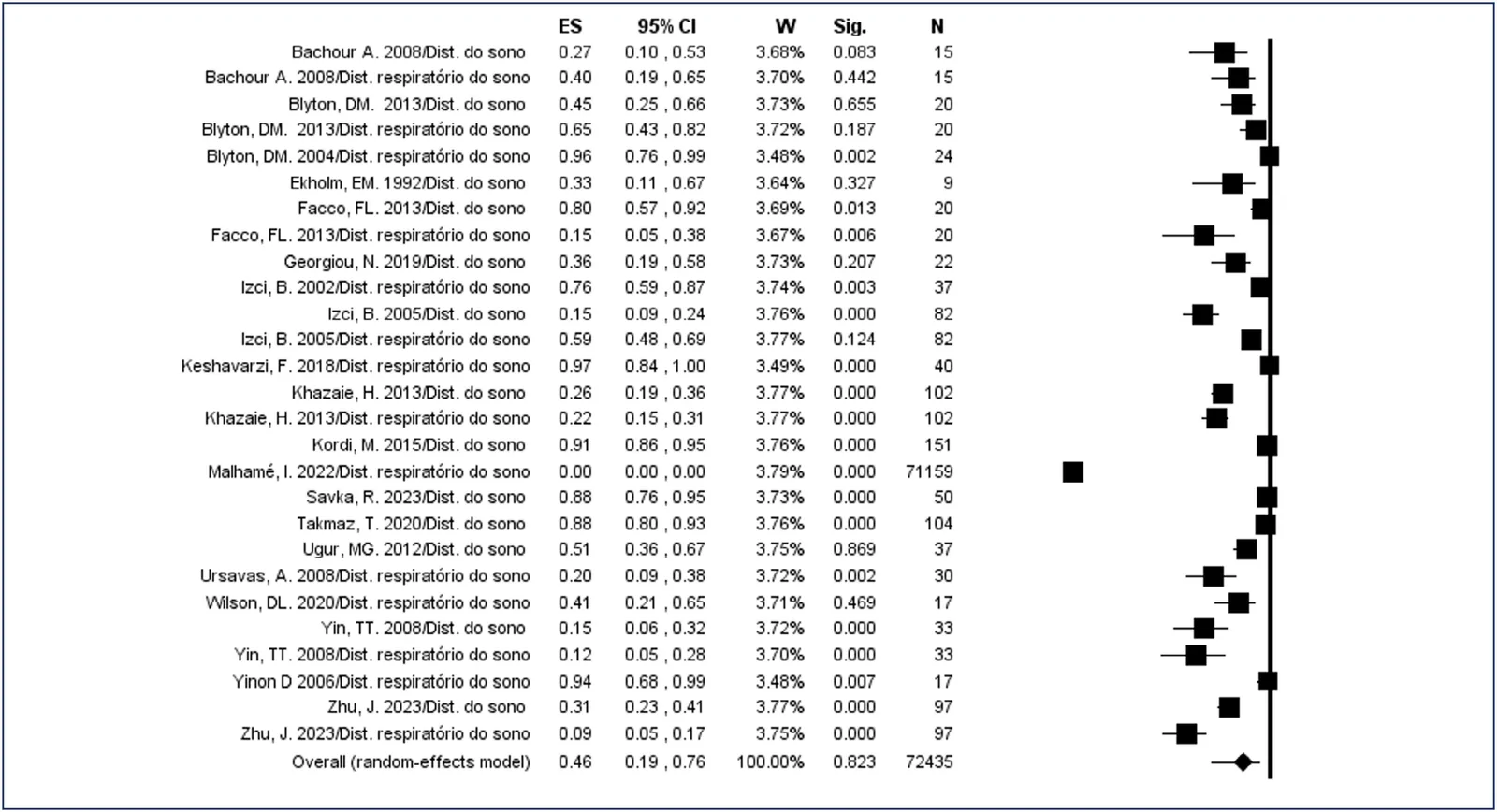
Forest plot: pooled prevalence of sleep disturbances in women with preeclampsia

In the domain of subjective sleep quality, studies investigated parameters such as insomnia, excessive daytime sleepiness, non-restorative sleep, sleep fragmentation, total sleep duration, nocturnal awakenings, nighttime movements, and global self-assessed sleep quality, often measured using the Pittsburgh Sleep Quality Index (PSQI) or similar instruments. In some cases, emotional factors such as morning fatigue, sadness, and anxiety directly affecting perceived sleep quality—were also included. In this analysis, the pooled prevalence was 0.50 (95% CI [0.31–0.70]) (Figure 3), with high heterogeneity (I^2^ = 94.56%).

**Figure 3:**
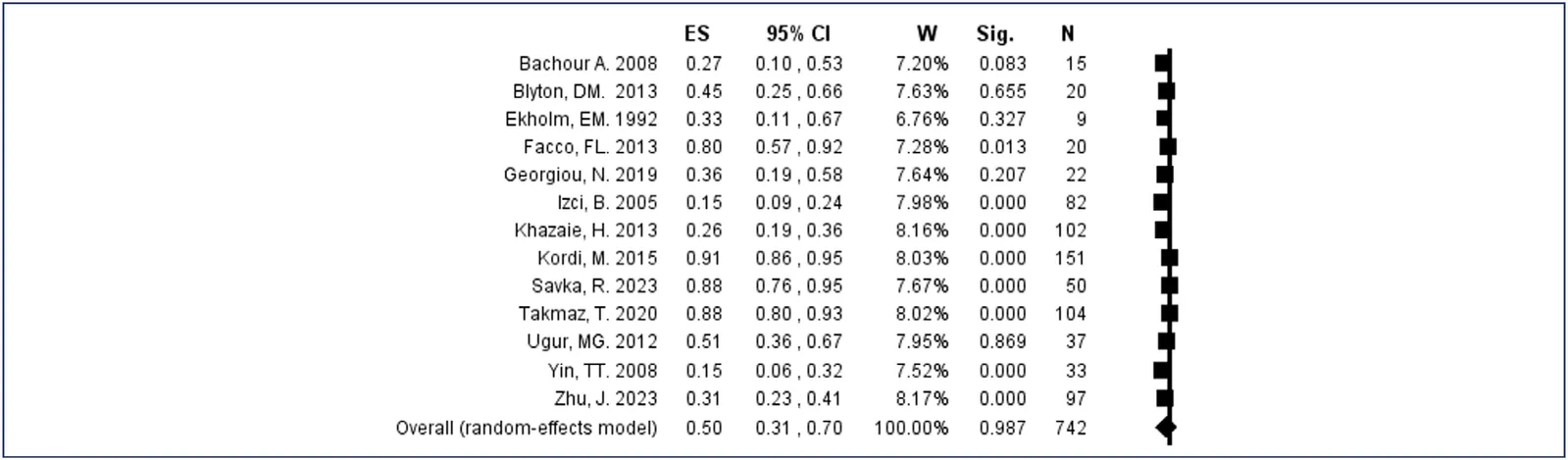
Forest plot: prevalence of qualitative sleep disturbances in preeclamptic women

In the category of respiratory sleep disorders, variables assessed included habitual snoring, obstructive sleep apnea (OSA), upper-airway flow limitation, apnea–hypopnea index (AHI), oxygen desaturations, and reduced REM sleep duration. These disturbances were identified through both self-report (questionnaires) and objective methods, such as polysomnography and portable respiratory monitoring devices (e.g., Watch-PAT100, GoWatch). The pooled prevalence for this category was 0.43 (95% CI [0.10–0.83]) (Figure 4), with very high heterogeneity (I^2^ = 99.24%).

**Figure 4:**
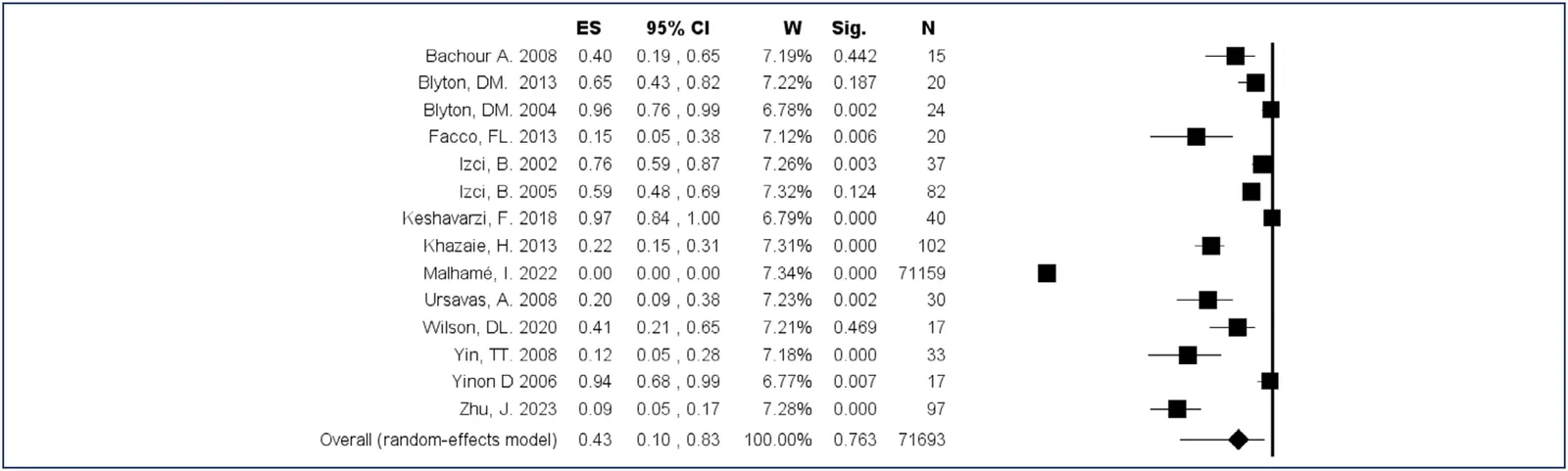
Forest plot: prevalence of sleep-disordered breathing in women with preeclampsia

A univariable meta-regression was performed to investigate potential sources of the substantial heterogeneity observed in the main analysis. The variables explored included publication year (Figure 5) and sample size (Figure 6). Neither variable accounted for the extreme heterogeneity (β ≈ 0, p > 0.05), and the inclusion of these moderators did not reduce heterogeneity (R^2^ ≈ 0%).

**Figure 5:**
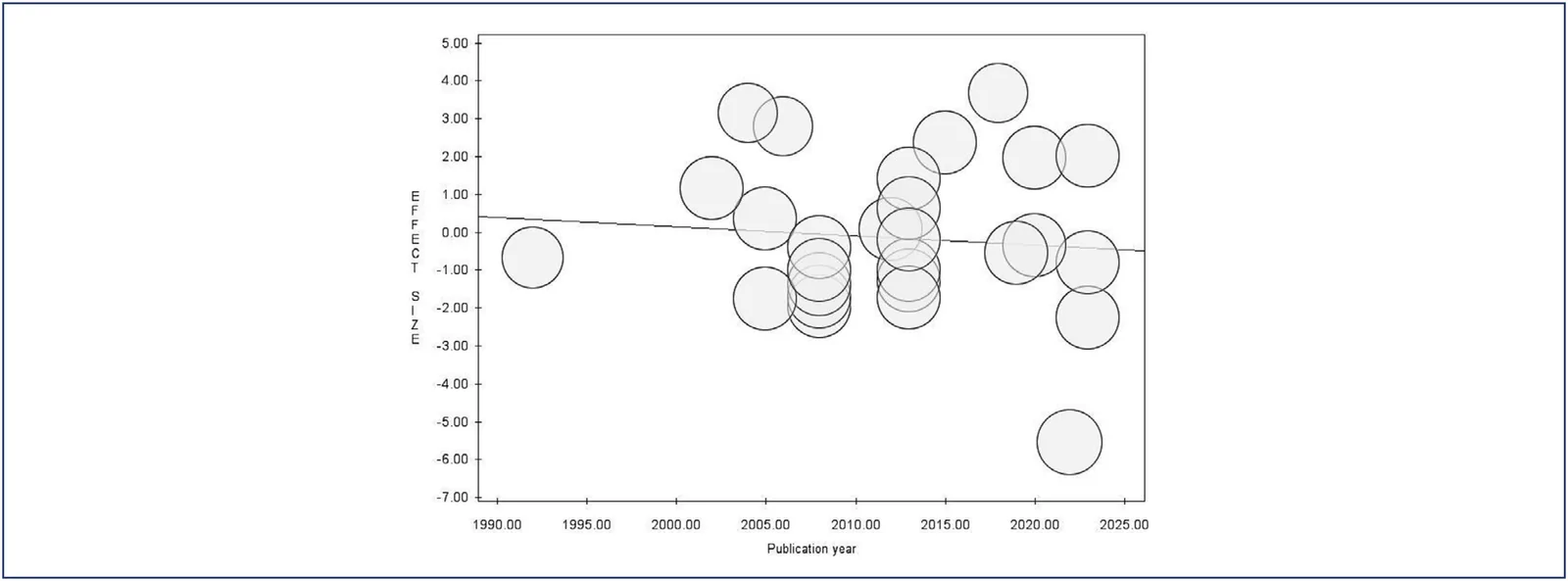
Meta-regression assessing the influence of publication year on overall prevalence

**Figure 6:**
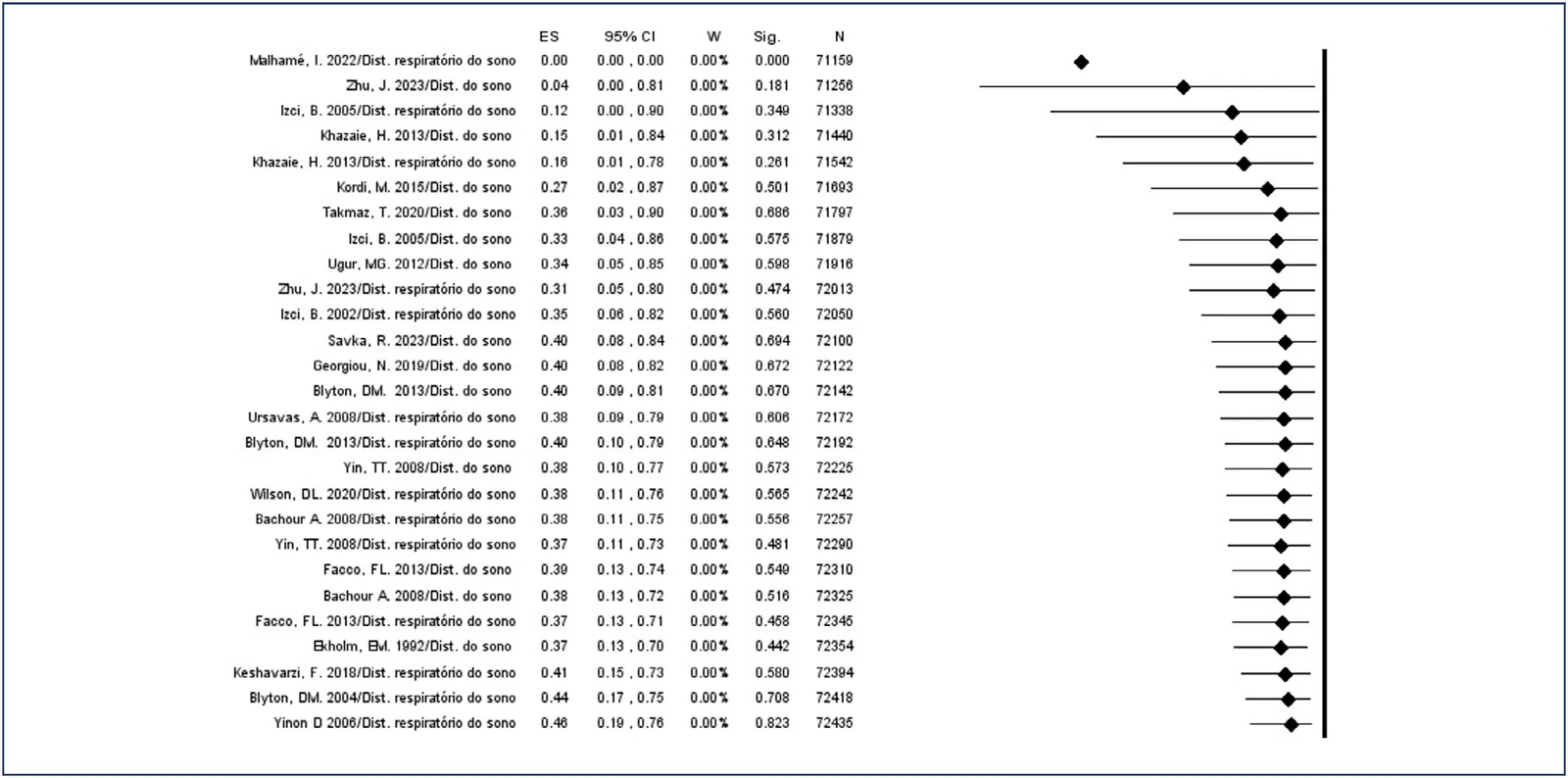
Meta-regression assessing the influence of sample size on overall prevalence

### Risk of bias assessment

Overall, the methodological quality of the included studies was moderate, with selection and confounding biases representing the most significant limitations (Figure 7).

**Figure 7:**
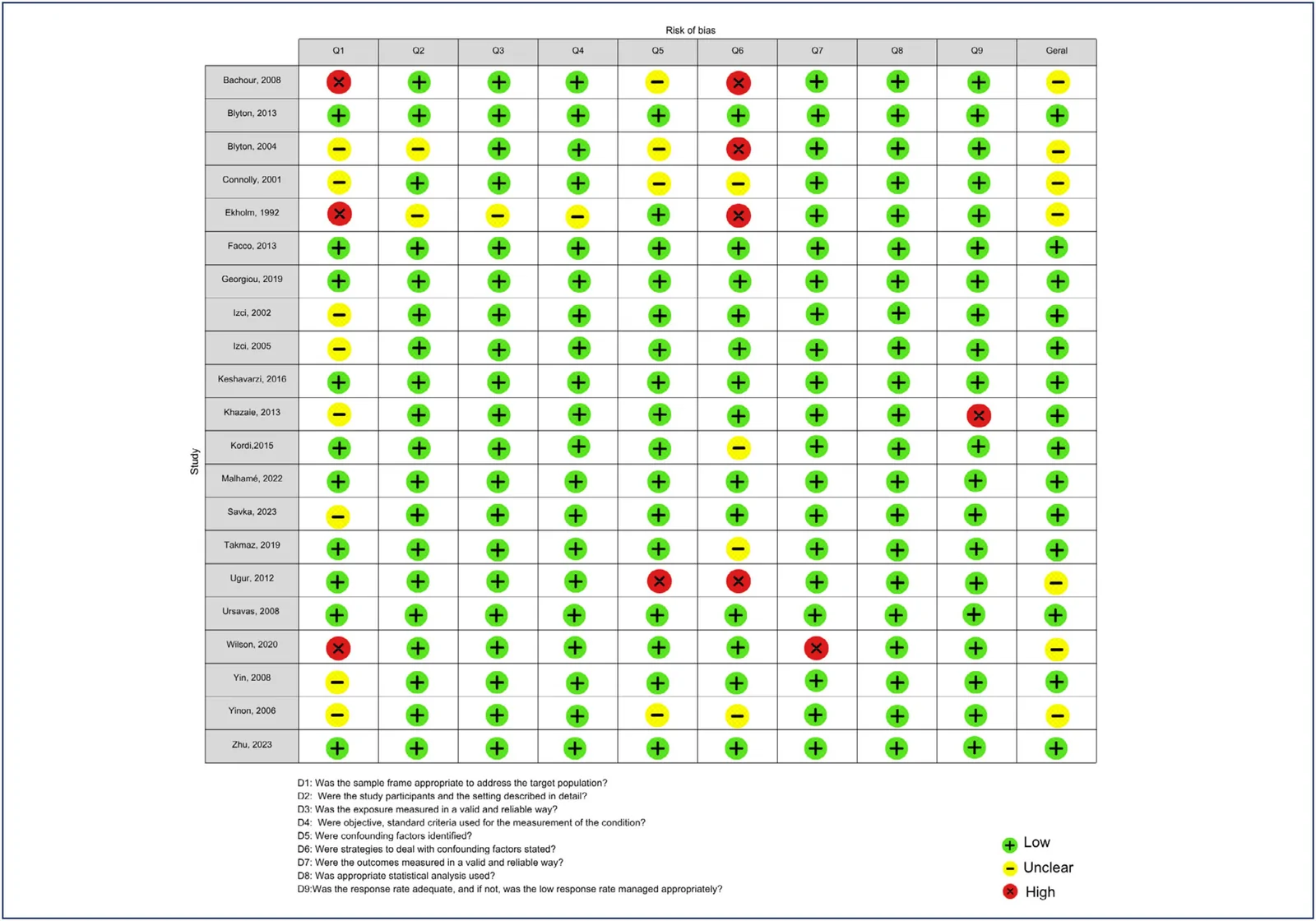
Risk of bias assessment: Joanna Briggs Institute (JBI) critical appraisal tool results for included studies

The strength of the evidence, according to the *Grading of Recommendations, Assessment, Development and Evaluation* (GRADE), was rated as very low. The certainty of evidence was downgraded due to: risk of bias (the majority of studies were observational; 40% had moderate or high risk of bias according to JBI), inconsistency (I^2^ > 99%, with extreme variability between studies), indirectness (outcomes measured with multiple non-standardized tools such as PSQI, self-report, and polysomnography), and imprecision (several confidence intervals crossing the null). Assessment of publication bias demonstrated asymmetry in the funnel plot (Figure 8). Egger's test indicated statistically significant asymmetry (p < 0.001), as did Begg's test (p = 0.015). However, the trim-and-fill analysis did not impute additional studies or modify the pooled estimate, suggesting no detectable missing studies based on this method. Considering the extreme heterogeneity among studies (I^2^ = 99.27%), the observed asymmetry should be interpreted with caution, as it may reflect true variability across populations and methodologies rather than publication bias alone.

**Figure 8:**
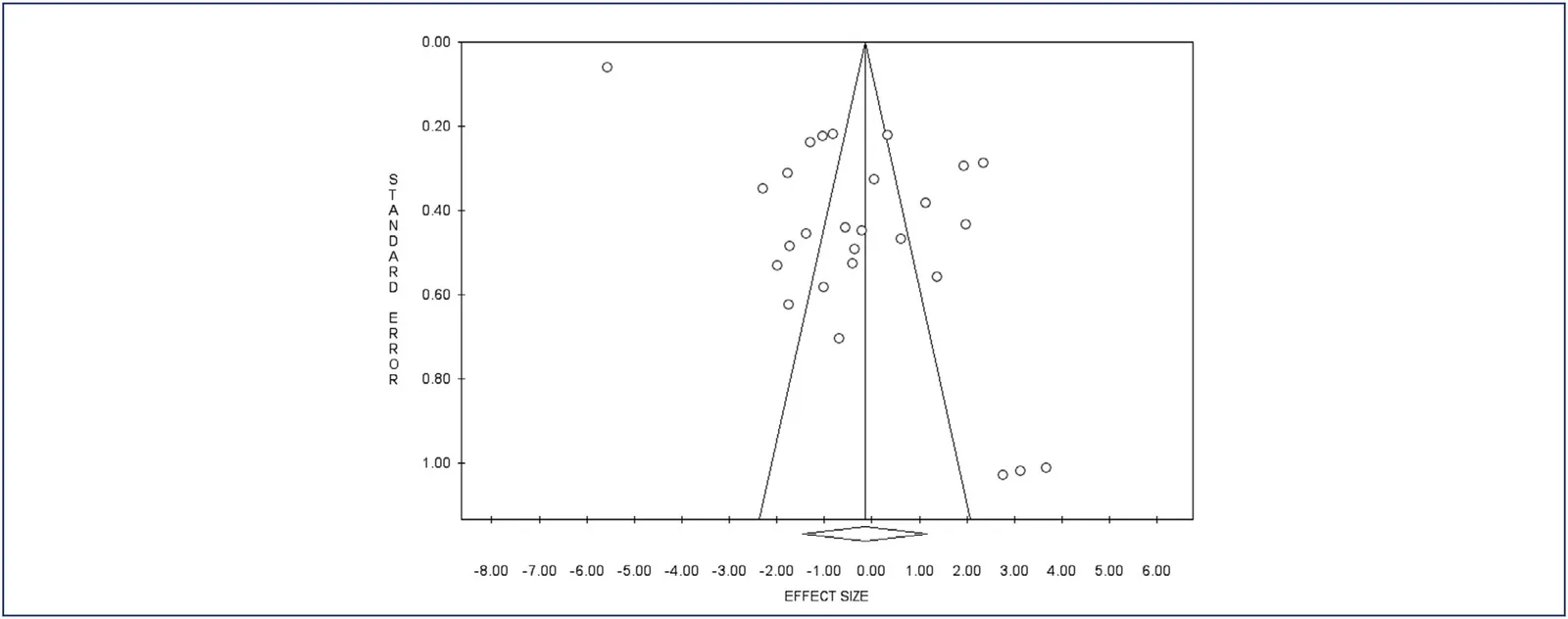
Funnel plot assessing publication bias in the meta-analysis of the prevalence of sleep disorders among pregnant women with preeclampsia

## Discussion

Our meta-analysis revealed a pooled prevalence of 46% (95% CI: 19% to 76%) of sleep disorders among women diagnosed with preeclampsia; however, this estimate was not statistically significant (p = 0.823). The lack of statistical significance is largely explained by the extremely high heterogeneity (I^2^ = 99.27%) across the included studies, which inflated the standard error of the combined estimate. It is important to emphasize that this study focuses on estimating the prevalence of sleep disturbances in this specific population, rather than establishing a causal association between sleep disturbances and preeclampsia. Nonetheless, the prevalence is clinically relevant, indicating that nearly half of pregnant individuals with preeclampsia may experience some type of sleep disturbance during pregnancy, consistent with the inflammatory and hemodynamic pathophysiology of preeclampsia, which may exacerbate insomnia, apnea, and sleep fragmentation.^(38)^

The strength of evidence for the pooled prevalence of sleep disorders in pregnant women with preeclampsia was rated as very low according to GRADE criteria. This classification arises from several factors. First, a notable risk of bias was observed, as most included studies were observational and exhibited moderate to high risk of bias, especially due to shortcomings in sample selection and outcome measurement, as assessed by the JBI tool. In addition, indirectness was present due to substantial variability in the instruments used to assess sleep disorders (e.g., self-reported PSQI versus polysomnography) and differences in diagnostic criteria for preeclampsia, both of which limit generalizability. Imprecision also weakened the evidence, reflected in the extremely wide confidence interval (19% to 76%), indicating substantial uncertainty in the true prevalence. Moreover, statistical heterogeneity was extreme (I^2^ = 99.27%), representing the major limitation and suggesting that the pooled estimate may not reflect a single underlying prevalence across the population studied. Thus, the p-value of 0.823 does not suggest the absence of sleep disturbances but rather highlights the uncertainty and variability surrounding the global estimate, making the combined prevalence insufficiently reliable for precise inference.

The substantial heterogeneity observed (I^2^ = 99.27%) warranted an in-depth exploration of its potential sources. Exploratory meta-regression analyses did not identify significant predictors capable of explaining this variability. Nonetheless, several plausible contributors may have influenced the variability across studies. Some heterogeneity may derive from methodological differences in sleep assessment, as the use of disparate instruments (self-report questionnaires such as the PSQI versus objective tools like polysomnography) can yield different prevalence estimates. Furthermore, population characteristics—including mean age, body mass index, parity, ethnicity, and gestational trimester at assessment—may considerably influence prevalence. Variation in diagnostic criteria for preeclampsia (e.g., ACOG vs. ISSHP definitions) may also affect comparability across studies, as these criteria shape the clinical composition of samples. Additionally, the operational definition of "sleep disorder" varied, with some studies grouping insomnia, obstructive apnea, excessive sleepiness, or restless legs syndrome without adequate stratification, further inflating variability. The persistence of unexplained heterogeneity suggests that the true prevalence may vary meaningfully across clinical and population contexts, limiting the generalizability of a single combined estimate.

The relationship between sleep disorders and preeclampsia is physiologically plausible and aligned with the mechanisms described in the introduction. Systemic inflammation and metabolic dysregulation, such as insulin resistance and dyslipidemia, are common consequences of sleep disturbances and well-established risk factors for preeclampsia. Sleep disorders are prevalent during pregnancy and have been associated with increased systemic inflammation, insulin resistance, and dyslipidemia.^(10;42-44)^ Sleep deprivation and sleep-disordered breathing activate proinflammatory pathways, increase circulating cytokines, and promote sympathetic nervous system activity, contributing to endothelial dysfunction and metabolic syndrome features,^(44-47)^ all of which play key roles in the pathogenesis of preeclampsia. Women with obesity, diabetes, or sleep disorders exhibit higher levels of proinflammatory cytokines, altered glycemic profiles, and abnormal lipid metabolism, all of which increase preeclampsia risk.^(45;48-50)^ Sleep disturbances may further elevate preeclampsia risk by impairing vascular function, promoting sympathetic hyperactivity, and exacerbating metabolic dysregulation.^(44-47)^ Epidemiological and mechanistic studies support the causal relationship among sleep disturbances, metabolic dysfunction, and adverse pregnancy outcomes, although important gaps remain. Improving sleep quality and treating sleep-disordered breathing may enhance metabolic status and potentially reduce risk, but additional studies are needed to clarify the direct mechanistic pathways during pregnancy.^(42,44,47)^ Therefore, although the included studies did not directly explore these mechanisms, our findings of high prevalence underscore the need for future investigations to elucidate the molecular and cellular pathways underlying this co-occurrence and to address existing knowledge gaps.

When compared with the existing literature, the pooled prevalence of 46% for sleep disorders in pregnant individuals with preeclampsia is notably higher than that reported in meta-analyses of general pregnant populations, in which prevalence tends to be lower. Meta-analyses in general obstetric populations report prevalence rates of insomnia symptoms ranging from 38–44%, poor sleep quality from 34–46% depending on trimester, and sleep-disordered breathing from 4–20%.^(51-55)^ For example, one meta-analysis found a prevalence of poor sleep quality (PSQI ≥ 5) of 45.7% during pregnancy, but this estimate includes all pregnant individuals, not specifically those at high risk or with preeclampsia.^(54)^ In contrast, studies focused on high-risk groups, such as individuals with preeclampsia or other risk factors, consistently report higher prevalence rates of sleep disturbances, with combined rates reaching or exceeding 46%.^(17)^ This difference underscores the increased burden of sleep disturbances among women with preeclampsia and other high-risk pregnancies compared with the general obstetric population.^(17,52,54,55)^

The high prevalence of sleep disorders in preeclampsia supports the hypothesis that preeclampsia may be both a risk factor for and a marker of sleep disturbances in high-risk pregnancies, although heterogeneity and confounders warrant further investigation. Several systematic reviews and meta-analyses consistently demonstrate a strong association between sleep disturbances and preeclampsia, with prevalence rates in high-risk groups (such as those with preeclampsia) surpassing those of the general obstetric population.^(10,11,17;56-59)^ Prospective cohort studies and Mendelian randomization analyses further suggest bidirectional relationships: preeclampsia may predispose individuals to sleep disorders due to shared pathophysiological mechanisms (e.g., systemic inflammation, metabolic dysregulation, vascular dysfunction), while sleep disturbances independently increase the risk of developing preeclampsia.^(12,15,17,18,57,60,61)^ Polysomnographic and questionnaire-based studies show that sleep-disordered breathing and insomnia are more severe and more frequent in women with preeclampsia, even after adjustment for confounders such as BMI and maternal age.^(12,57,60)^ Overlapping risk factors including obesity, advanced maternal age, and metabolic syndrome further support the notion that preeclampsia is both a risk factor and a marker of sleep disorders in high-risk pregnancies.^(17,18,61)^ Thus, despite considerable heterogeneity in study design, populations, and diagnostic criteria, the consistently elevated prevalence and severity of sleep disturbances in preeclampsia reinforce the hypothesis that preeclampsia serves as both a risk factor and a marker of sleep disorders in high-risk pregnancies. However, additional research is needed to clarify causality, account for confounding, and guide optimal screening and management strategies.^(10,11,18,59,61)^ Accordingly, this study contributes to the body of knowledge by synthesizing data specific to pregnant individuals with preeclampsia, addressing a gap in the literature and highlighting the need for appropriate screening and management in this population.

This systematic review and meta-analysis has several important limitations that should be considered when interpreting the results. First, the risk of bias in the primary studies, assessed using the JBI tool, was predominantly moderate to high due to the observational nature of the studies and the lack of blinding or control for confounders, which may have led to overestimation or underestimation of prevalence. Second, variability in the instruments used to assess sleep disturbances (e.g., self-report questionnaires such as the PSQI versus objective methods such as polysomnography) may have introduced heterogeneity and complicated direct comparisons across studies. Reliance on self-reported data in many studies may either underestimate or overestimate the true prevalence. Third, the absence of standardized data on specific subgroups (e.g., severity of preeclampsia, gestational age at diagnosis, presence of comorbidities) prevented more refined analyses that might have explained some heterogeneity and yielded more precise estimates for specific populations. Additionally, although we conducted a comprehensive search, only three electronic databases (MEDLINE, Embase, and LILACS) were included, which may have limited the identification of all relevant studies. Finally, although Egger's and Begg's tests indicated statistically significant funnel-plot asymmetry, the trim-and-fill analysis did not identify missing studies nor alter the pooled estimate. This pattern suggests that the asymmetry observed is likely attributable to extreme heterogeneity, differences in study design, sleep assessment tools, and diagnostic criteria for preeclampsia, rather than classical publication bias. Thus, consistent evidence of publication bias compromising interpretation is lacking, although the inability to fully assess robustness leaves open the possibility that true prevalence may be overestimated if null or negative studies were not published. Collectively, these limitations contribute to the low confidence in the pooled estimate, as reflected in the GRADE assessment, and should be taken into account when evaluating the clinical applicability of our findings.

These findings have important implications for clinical practice and future research. Given the high prevalence of sleep disturbances among individuals with preeclampsia, even in the context of uncertainty surrounding the pooled estimate, it is prudent to consider screening for sleep disorders in this high-risk population. Early identification may enable nonpharmacological interventions that improve maternal quality of life and potentially pregnancy outcomes. For future research, the following directions are priorities for strengthening the evidence base: (1) Prospective longitudinal studies: essential for establishing temporal and causal relationships between sleep disturbances and preeclampsia, using objective sleep assessment methods (e.g., polysomnography) and standardized diagnostic criteria for both conditions; (2) Individual participant data (IPD) meta-analyses: which would allow more robust examination of heterogeneity by adjusting for individual-level characteristics and standardizing outcomes, overcoming limitations of aggregate data; (3) Mechanistic studies: to investigate the specific physiological pathways linking sleep disturbances to preeclampsia, including inflammatory and metabolic biomarkers, in order to identify therapeutic targets; and (4) Randomized clinical trials: to assess the effectiveness of interventions for sleep disorders in preventing or managing preeclampsia, using relevant maternal and neonatal outcomes. Implementing these recommendations is critical for strengthening the evidence base and informing future clinical guidelines aimed at improving maternal and infant health.

## Conclusion

This meta-analysis identified an estimated prevalence of 46% of sleep disorders among individuals with preeclampsia; however, the certainty of the evidence was rated as very low according to GRADE, primarily due to extreme heterogeneity and uncontrolled risk of bias. Future research is warranted to evaluate the cost–benefit of screening for sleep disorders in pregnant women with preeclampsia. Such studies should standardize diagnostic criteria for both preeclampsia and sleep disorders.

## Data Availability

The research data are described in the article presented.
