# Supplementary material for: Sleep patterns and sleep disorders in pregnant women with preeclampsia: a systematic review and meta-analysis
Source: Rev Bras Ginecol Obstet. 2026 Aug 20;48:e-rbgo54. doi: 10.61622/rbgo/2026rbgo54 (PMC13690384; doi:10.61622/rbgo/2026rbgo54)
Supplement: Supplementary file 1 [file 1806-9339-rbgo-48-e-rbgo54-Suppl01.pdf]

Table 1S. Search strategy applied to each database (PubMed, Embase, and LILACS)

Databases	Descriptors
PubMed	#1 Pre Eclampsia OR Preeclampsia OR Pregnancy Toxemias OR Pregnancy Toxemia OR Toxemia, Pregnancy OR Edema-Proteinuria-Hypertension Gestosis OR Edema Proteinuria Hypertension Gestosis OR Gestosis, Edema-Proteinuria-Hypertension OR Hypertension-Edema-Proteinuria Gestosis OR Gestosis, Hypertension-Edema-Proteinuria OR Hypertension Edema Proteinuria Gestosis OR Proteinuria-Edema-Hypertension Gestosis OR Gestosis, Proteinuria-Edema-Hypertension OR Proteinuria Edema Hypertension Gestosis OR EPH Complex OR EPH Toxemias OR EPH Toxemia OR Toxemia, EPH OR Toxemias, EPH OR EPH Gestosis OR Gestosis, EPH OR Toxemias, Pregnancy OR Toxemia Of Pregnancy OR Of Pregnancies, Toxemia OR Of Pregnancy, Toxemia OR Pregnancies, Toxemia Of OR Pregnancy, Toxemia Of OR Toxemia Of Pregnancies OR Preeclampsia Eclampsia 1 OR 1, Preeclampsia Eclampsia OR 1s, Preeclampsia Eclampsia OR Eclampsia 1, Preeclampsia OR Eclampsia 1s, Preeclampsia OR Preeclampsia Eclampsia<br>55.508<br><br>#2 Disorder, Sleep Wake OR Disorders, Sleep Wake OR Sleep Wake Disorder OR Wake Disorder, Sleep OR Wake Disorders, Sleep OR Sleep Disorders OR Disorder, Sleep OR Disorders, Sleep OR Sleep Disorder OR Long Sleeper Syndrome OR Long Sleeper Syndromes OR Sleeper Syndrome, Long OR Sleeper Syndromes, Long OR Syndrome, Long Sleeper OR Syndromes, Long Sleeper OR Short Sleeper Syndrome OR Short Sleeper Syndromes OR Sleeper Syndrome, Short OR Sleeper Syndromes, Short OR Syndrome, Short Sleeper OR Syndromes, Short Sleeper OR Short Sleep Phenotype OR Phenotype, Short Sleep OR Phenotypes, Short Sleep OR Short Sleep Phenotypes OR Sleep Phenotypes, Short OR Sleep-Related Neurogenic Tachypnea OR Neurogenic Tachypnea, Sleep-Related OR Neurogenic Tachypneas, Sleep-Related OR Sleep Related Neurogenic Tachypnea OR Sleep-Related Neurogenic Tachypneas OR Tachypnea, Sleep-Related Neurogenic OR Tachypneas, Sleep-Related Neurogenic OR Subwakefullness Syndrome OR Subwakefullness Syndromes OR Syndromes, Subwakefullness OR Syndrome, Subwakefullness<br>177,352<br><br>#3 Deprivation, Sleep OR Insufficient Sleep Syndrome OR Insufficient Sleep Syndromes OR Syndrome, Insufficient Sleep OR REM Sleep Deprivation OR Deprivation, REM Sleep OR Sleep Deprivation, REM OR Sleep Fragmentation OR Fragmentation, Sleep OR Insufficient Sleep OR Sleep, Insufficient OR Inadequate Sleep OR Sleep, Inadequate OR Sleep Debt OR Sleep Insufficiency OR Insufficiencies, Sleep OR Insufficiency, Sleep OR Sleep Insufficiencies<br>26.921<br><br>#4 Duration, Sleep OR Sleep Quantity OR Quantity, Sleep OR Sleep Quantities OR Total Sleep Time<br>44.572<br><br>#5 Qualities, Sleep OR Quality, Sleep OR Sleep Qualities<br>54.673<br><br>#6 Latency, Sleep OR Sleep Latencies OR Sleep Onset Latency OR Latency, Sleep Onset OR Onset Latencies, Sleep OR Sleep Onset Latencies OR REM Sleep Latency OR Latency, REM Sleep OR REM Sleep Latencies OR Sleep Latency, REM<br>10.504<br><br>#1 AND #2<br>1.690<br><br>#1 AND #3<br>26<br><br>#1 AND #4<br>41<br><br>#1 AND #5<br>43<br><br>#1 AND #6<br>4
Embase	#1 'preeclampsia'/exp OR 'preeclampsia'<br>91,347<br><br>#2 'sleep deprivation'<br>24,566<br><br>#3 'sleep time'<br>48,031<br><br>#4 'sleep quality'<br>59,706<br><br>#5 'sleep latency'<br>13,961<br><br>#1 AND #2<br>33<br>#1 AND #3<br>84<br><br>#1 AND #4<br>100<br><br>#1 AND #5<br>8

Continue...

Continuation.

Databases	Descriptors
LILACS	#1 Pre Eclampsia OR Preeclampsia OR Pregnancy Toxemias OR Pregnancy Toxemia OR Toxemia, Pregnancy OR Edema-Proteinuria-Hypertension Gestosis OR Edema Proteinuria Hypertension Gestosis OR Gestosis, Edema-Proteinuria-Hypertension OR Hypertension-Edema-Proteinuria Gestosis OR Gestosis, Hypertension-Edema-Proteinuria OR Hypertension Edema Proteinuria Gestosis OR Proteinuria-Edema-Hypertension Gestosis OR Gestosis, Proteinuria-Edema-Hypertension OR Proteinuria Edema Hypertension Gestosis OR EPH Complex OR EPH Toxemias OR EPH Toxemia OR Toxemia, EPH OR Toxemias, EPH OR EPH Gestosis OR Gestosis, EPH OR Toxemias, Pregnancy OR Toxemia Of Pregnancy OR Of Pregnancies, Toxemia OR Of Pregnancy, Toxemia OR Pregnancies, Toxemia Of OR Pregnancy, Toxemia Of OR Toxemia Of Pregnancies OR Preeclampsia Eclampsia 1 OR 1, Preeclampsia Eclampsia OR 1s, Preeclampsia Eclampsia OR Eclampsia 1, Preeclampsia OR Eclampsia 1s, Preeclampsia OR Preeclampsia Eclampsia 1s<br>1.530<br><br>#2 Deprivation, Sleep OR Insufficient Sleep Syndrome OR Insufficient Sleep Syndromes OR Syndrome, Insufficient Sleep OR REM Sleep Deprivation OR Deprivation, REM Sleep OR Sleep Deprivation, REM OR Sleep Fragmentation OR Fragmentation, Sleep OR Insufficient Sleep OR Sleep, Insufficient OR Inadequate Sleep OR Sleep, Inadequate OR Sleep Debt OR Sleep Insufficiency OR Insufficiencies, Sleep OR Insufficiency, Sleep OR Sleep Insufficiencies<br>11.709<br><br>#3 Duration, Sleep OR Sleep Quantity OR Quantity, Sleep OR Sleep Quantities OR Total Sleep Time<br>13.655<br><br>#4 Qualities, Sleep OR Quality, Sleep OR Sleep Qualities<br>60.748<br><br>#5 Latency, Sleep OR Sleep Latencies OR Sleep Onset Latency OR Latency, Sleep Onset OR Onset Latencies, Sleep OR Sleep Onset Latencies OR REM Sleep Latency OR Latency, REM Sleep OR REM Sleep Latencies OR Sleep Latency, REM<br>565<br><br>#1 AND #2<br>1<br><br>#1 AND #3<br>1<br><br>#1 AND #4<br>0<br><br>#1 AND #5<br>0
